# Finite Element Model Updating for a Continuous Beam-Arch Composite Bridge Based on the RSM and a Nutcracker Optimization Algorithm

**DOI:** 10.3390/s25154831

**Published:** 2025-08-06

**Authors:** Weihua Zhou, Hongyin Yang, Jing Hao, Mengxiang Zhai, Hongyou Cao, Zhangjun Liu, Kang Wang

**Affiliations:** 1 School of Civil Engineering and Architecture, Wuhan Institute of Technology, Wuhan 430073, China; 2 State Key Laboratory of Bridge Intelligent and Green Construction, Wuhan 430034, China; 3 School of Civil Engineering and Architecture, Wuhan University of Technology, Wuhan 430070, China

**Keywords:** continuous beam–arch composite bridge, finite element model updating, response surface method, nutcracker optimization algorithm, stochastic subspace identification

## Abstract

Accurate finite element (FE) models are essential for the safety assessment of civil engineering structures. However, obtaining reliable model parameters for existing bridges remains challenging due to the inability to conduct static load tests without disrupting traffic flow. To address this, this study proposes an FE model updating framework that integrates the response surface method and the nutcracker optimization algorithm (NOA). This framework is characterized by the incorporation of ambient vibration data into parameter optimization, thereby enhancing model accuracy. The stochastic subspace identification method is first adopted to extract the bridge’s natural frequencies from vibration data. The response surface method is then employed to construct a response surface function that approximates the FE model. The NOA is subsequently applied to iteratively optimize this response surface function, ensuring rapid convergence and the precise adjustment of the FE model parameter. To validate the effectiveness of the proposed framework, a continuous beam–arch composite bridge with a span of 204.783 m was selected as a case study. The results indicate that the proposed method reduced the average frequency error from 5.58% to 2.75% by updating the model parameters. While the whale optimization algorithm required 21 iterations and the grey wolf optimizer needed 41 iterations to converge near the minimum, the NOA achieved this in merely 13 iterations, demonstrating the NOA’s superior convergence speed. Furthermore, the NOA significantly outperformed both the whale optimization algorithm and the grey wolf optimizer in reducing the error of the first transverse vibration frequency.

## 1. Introduction

Finite element (FE) modeling is universally acknowledged as the foundation of contemporary structural analysis [1]. The developed FE models, however, often deviate significantly from the corresponding physical structures, especially when accounting for material degradation, connection nonlinearity, and environmental variability [2]. These inaccuracies inevitably propagate through structural performance predictions, ultimately undermining safety assessments and lifecycle management strategies. This disparity underscores an imperative need for developing scientifically rigorous and computationally viable model updating techniques to close the gap between virtual simulations and physical realities [3,4].

Based on the type of FE model, model-updating methods can be classified into direct model updating and surrogate-based model updating. Due to drawbacks such as difficulties in parameter selection, direct model updating has not been widely adopted [5]. Consequently, surrogate models have gained widespread adoption in engineering practice, primarily due to their superior computational efficiency [6]. Wang Chengwei et al. utilized long short-term memory networks as a surrogate model for bridge structures to achieve FE model parameter updating [7], while Haifang He et al. employed wavelet neural networks as a surrogate model for a continuous beam structure of three equal spans [8]. Beyond neural networks serving as surrogate models for FEs, the response surface model (RSM) [9,10,11] and the Kriging model [12] are also frequently employed. The response surface method, which approximates implicit relationships between structural parameters and system responses through low-order functions [6,13], can significantly reduce computational costs during the model updating process [14]. Xia Zhiyuan et al. employed sensitivity analysis to select 13 variables as parameters for the response surface function, took the first five natural frequencies and vertical displacements of the main girder as target values, and established second-order response surface expressions for a continuous beam–arch composite bridge [15]. Ji Wei and Shao Tianyan used the concrete mass density, the elastic modulus of the segment 10 web, and the elastic modulus of the segment 15 steel bottom flange as response surface function parameters, took the fundamental bending frequency, mid-span deflection, and strain as target values, and constructed a quadratic response surface function for a novel corrugated-steel-web box-girder bridge [16]. Ren Weixin and Chen Huabing adopted a central composite design to determine RSM parameters, used the first five natural frequencies as response surface function targets, and built an FE model for a simply supported beam bridge [17].

With the increasing complexity of modern bridges, model iteration necessitates the optimization of high-dimensional parameters [18,19]. For engineering optimization problems featuring high parameter dimensionality and strong nonlinear relationships, traditional optimization algorithms are prone to becoming trapped in local optima. Consequently, the selection of optimization algorithms becomes a crucial aspect. Among these, genetic algorithms [20,21,22] and particle swarm optimization algorithms [23,24] have found widespread application within FE contexts. In recent years, an increasing number of scholars have applied metaheuristic optimization algorithms to identify optimal solutions for FE model parameters. Kang Juntao et al. combined an improved artificial fish swarm algorithm with Kriging models, obtained both global and local optima, and demonstrated through FE validation that local optima show superior consistency with actual structures compared to global solutions [25]. Wu Jie et al. proposed a parametric updating framework that synthesize Kriging models with intelligent optimization algorithms, revealing differential performance among the genetic algorithm, the bird mating optimization algorithm and the particle swarm optimization algorithm. During parameter searches, the particle swarm optimization algorithm achieved superior accuracy, while the genetic algorithm delivered faster computation when evaluating temporal efficiency [26]. Qin Shiqiang et al. combined Kriging models with metaheuristic algorithms, contrasting up to 11 distinct algorithms during finite FE updating for truss and cable-stayed bridges, concluding that improved algorithms deliver significantly higher precision [27].

This study proposes a finite element model updating method that combines the RSM and the NOA to improve the accuracy of structural dynamic characteristics for continuous beam–arch composite bridges under environmental excitation. The methodology begins with establishing the theoretical framework, integrating stochastic subspace identification (SSI) for modal parameter extraction, the RSM for surrogate model construction, and the NOA for parameter optimization. An engineering case study demonstrates implementation through the development of a detailed finite element model in ABAQUS, experimental modal analysis using SSI techniques, systematic construction and validation of the RSM surrogate model, and iterative NOA optimization to minimize frequency discrepancies between numerical and experimental results. This comprehensive approach provides an effective solution for finite element model updating of beam–arch bridge structures while maintaining computational efficiency through the combined RSM–NOA framework.

## 2. Materials and Methods

### 2.1. Identification of the Structural Natural Frequency

In practical engineering, bridges are often subjected to complex environmental excitations (such as wind loads and traffic loads) [28,29], and their dynamic characteristics need to be identified through operational modal analysis (OMA) [30]. Unlike traditional experimental modal analysis, OMA does not require known excitation input and can extract modal parameters based solely on environmental vibration responses [31], making it particularly suitable for complex structures [32,33]. This section briefly describes the SSI method in OMA.

Due to the fact that the response data measured by sensors are usually discrete and cannot meet the requirements of continuity, the continuous state equation and dynamic equation of the structural system are transformed into discrete equations, as shown below:

(1) $$\left\{\begin{array}{l} x_{k+1}=Ax_{k}+w_{k}\\ y_{k}=Cx_{k}+v_{k} \end{array}\right.$$

where A represents the state matrix of the system, C represents the output matrix of the system, $x_{k}$ and $y_{k}$, respectively, represent the state vector and output vector of the system at discrete time k, and $w_{k}$ and $v_{k}$, respectively, represent the process noise and measurement noise of the system at time k. From this, it can be seen that the state matrix and output matrix of the system are keys to identifying the modal parameters of the system.

The core principle of SSI is to project the “future” matrix onto the “past” matrix [26], as shown below:

(2) $$O_{i}=Y_{f}/Y_{p}=Y_{f}Y_{p}^{T}{\left(Y_{p}Y_{p}^{T}\right)}^{+}Y_{p}$$

where $Y_{p}$ is the “past” matrix of the Hankel matrix and $Y_{f}$ is the “future” matrix of the Hankel matrix, ${\left(*\right)}^{+}$ representing the generalized inverse of the matrix ∗.

Decompose the projection matrix $O_{i}$ into the product of the observable vector $\Gamma_{i}$ and the Kalman filter state vector ${\bar{X}}_{i}$, as shown below:

(3) $$O_{i}=\Gamma_{i}{\bar{X}}_{i}$$

Subsequently, perform singular value decomposition on the projection matrix $O_{i}$:

(4) $$O_{i}={USV}^{T}=\left(\begin{array}{cc} U_{1} & U_{2} \end{array}\right)\left(\begin{array}{cc} S_{1} & 0\\ 0 & S_{2} \end{array}\right)\left(\begin{array}{c} V_{1}^{T}\\ V_{2}^{T} \end{array}\right)=U_{1}S_{1}V_{1}^{T}$$

Combining Equations (3) and (4) yields:

(5) $$\left\{\begin{array}{l} \Gamma_{i}=U_{i}S_{1}^{1/2}\\ {\bar{X}}_{i}=\Gamma_{i}^{+}O_{i} \end{array}\right.$$

Moving the “past” matrix down one row towards the “future” matrix yields another partitioning method, where the projection matrix is defined as:

(6) $$O_{i}=Y_{f}^{-}/Y_{p}^{+}=\Gamma_{i-1}{\bar{X}}_{i+1}$$

where $Y_{f}^{-}$ represents the matrix after the “future” matrix $Y_{f}$ is moved up one row, and $Y_{p}^{+}$ represents the matrix after the “past” matrix $Y_{p}$ is moved down one row.

From Equation (5), it can be concluded that:
  (7) $${\bar{X}}_{i+1}=\Gamma_{i-1}^{+}O_{i-1}$$

According to Equations (5) and (7), the Kalman filter state vector ${\bar{X}}_{i}$ and ${\bar{X}}_{i+1}$ can be obtained by the system output response. By substituting ${\bar{X}}_{i}$ and ${\bar{X}}_{i+1}$ into Equation (2), we obtain:

(8) $$\left(\begin{array}{c} {\bar{X}}_{i+1}\\ Y_{i|i} \end{array}\right)=\left(\begin{array}{c} A\\ C \end{array}\right){\bar{X}}_{i}+\left(\begin{array}{c} W_{i}\\ V_{i} \end{array}\right)$$

where $W_{i}$ and $V_{i}$ are the residuals that are independent of ${\bar{X}}_{i}$, and $Y_{i|i}$ are the rows in the Hankel matrix.

In summary, by using the least squares method to solve Equation (8), the state matrix A of the system can be obtained, and eigenvalue decomposition can be performed on the state matrix to extract candidate frequency values from the eigenvalues. To determine whether the current frequency value constitutes a stable point [34], the following formula was applied to determine whether or not it is a stable point:

(9) $$\frac{\left|f_{\mathrm{curr}}-f_{\mathrm{prev}}\right|}{f_{\mathrm{curr}}}<0.02$$

where $f_{\mathrm{curr}}$ denotes the candidate frequency at the current order k, and $f_{\mathrm{prev}}$ represents the frequency value at the preceding order k−1.

### 2.2. Construction of a Surrogate Model Based on Response Surface Methodology

RSM, as a statistical regression technique [35], requires establishing a clear mathematical relationship between target response values and sensitive model parameters through response surface equations [36] in bridge FE model updating. This method first determines the parameters of the FE model to be updated, uses Box–Behnken experimental design to sample in the parameter space, generates a combination of FE model parameters, and calculates the output response of the FE model. We generated a fully quadratic polynomial to establish a parameter frequency RSM, and its mathematical expression is:

(10) $${\hat{f}}_{j}\left(x\right)=\beta_{0}^{\left(j\right)}+\sum_{i=1}^{k}\beta_{i}^{\left(j\right)}x_{i}+\sum_{i=1}^{k}\beta_{ii}^{\left(j\right)}x_{i}^{2}+\sum_{1<i<l<k}^{k}\beta_{il}^{\left(j\right)}x_{i}x_{l}$$

where k is the number of sensitive parameters, ${\hat{f}}_{j}$ is the predicted value of the j-th target response, $x_{i}$ represents the *i*-th FE model parameter to be updated, and $\beta_{0}^{\left(j\right)}$, $\beta_{i}^{\left(j\right)}$, $\beta_{ii}^{\left(j\right)}$, $\beta_{il}^{\left(j\right)}$ are regression coefficients obtained using the least squares method.

After obtaining the response surface function, we need to verify the prediction accuracy of the RSM at the sample points and test the fitting degree between the RSM and the sample points using the correlation coefficient $R^{2}$ and the root mean square error RMSE. The expressions for $R^{2}$ and RMSE are, respectively:

(11) $$R^{2}=1-\frac{\sum_{i=1}^{m}{\left({\hat{y}}_{i}-{y^{\prime }}_{i}\right)}^{2}}{\sum_{i=1}^{m}{\left({y^{\prime }}_{i}-\bar{y}\right)}^{2}}$$

(12) $$RMSE=\sqrt{\frac{1}{m}\sum_{i=1}^{m}{\left({\hat{y}}_{i}-{y^{\prime }}_{i}\right)}^{2}}$$

where ${\hat{y}}_{i}$ is the calculated value of the FE model corresponding to the *i*-th measured point, ${y^{\prime }}_{i}$ is the predicted response value of the *i*-th sample RSM, and $\bar{y}$ is the mean of the calculated values of finite FE models.

### 2.3. Parameter Optimization Process Based on the Nutcracker Optimization Algorithm

The NOA is a novel nature-inspired metaheuristic proposed by Mohamed et al. in 2023 [37]. Its design draws inspiration from the foraging behaviors of Clark’s nutcracker bird. During summer and autumn, the birds actively forage for pine seeds and subsequently cache them in suitable storage sites. Conversely, in winter and spring, they search for and retrieve these hidden caches by primarily relying on spatial memory. Nutcrackers utilize various environmental objects or landmarks as reference points to locate the stored seeds. When unable to locate a specific cache, they engage in random exploration of the search space to identify alternative food sources.

#### 2.3.1. Foraging Stage

- Exploration sub-stage: searching for pinecones in summer and autumn

At this stage, the nutcracker begins to select pinecones containing good pine nuts in the collection area. If the nutcracker does not find the desired pine nuts in the selected pine trees, the nutcracker will explore the good pine nuts in other pine trees in the collection area. The mathematical expression for the position update strategy at this stage is shown below:

(13) $${\vec{X}}_{i}^{t+1}=\left\{\begin{array}{ll} {\vec{X}}_{i}^{t} & \mathrm{if}\;\tau_{1}<\tau_{2}\\ \left\{\begin{array}{ll} {\vec{X}}_{m}^{t}+\lambda \cdot \left({\vec{X}}_{A}^{t}-{\vec{X}}_{B}^{t}\right)+\mu \cdot \left(r_{2}\cdot U-L\right) & \mathrm{if}\;t<T_{\max}/2\\ {\vec{X}}_{c}^{t}+\mu \cdot \left({\vec{X}}_{A}^{t}-{\vec{X}}_{B}^{t}\right)+\mu \cdot I\cdot \left(r^{2}\cdot U-L\right) & \end{array}\right. \end{array}\right.$$

where ${\vec{X}}_{i}^{t+1}$ is the new position of individual i at iteration t+1, ${\vec{X}}_{i}^{t}$ is the position of individual i at iteration t, λ is a random number generated by the Levy flight function, A and B are two randomly selected individuals from different nutcrackers from the population, U and L are the upper and lower limits for the parameter search, respectively, and ${\vec{X}}_{m}^{t}$ is the dimensional mean of all solutions of the current population in the iteration t. The value of μ is shown in the following equation:

(14) $$\mu =\left\{\begin{array}{l} \tau_{3}\;\;\;\mathrm{if}r_{1}<r_{2}\\ \tau_{4}\;\;\;\mathrm{if}r_{2}<r_{3}\\ \tau_{5}\;\;\;\mathrm{if}r_{1}<r_{3} \end{array}\right.$$

where $\tau_{4}$ is a number generated based on the normal distribution, $\tau_{5}$ is a random number generated by the Levy flight function, and $r_{1}$, $r_{2}$, $r_{3}$ are uniform random scalars within the range of $\left[0,1\right]$.

- Development sub-stage: pinecone storage

After completing the first stage of exploration and obtaining abundant pine nuts, the nutcracker uses the obtained pine nuts to store them far away from the collection area. The mathematical expression for the behavior of the nutcracker at this stage is shown in the equation:

(15) $${\vec{X}}_{i}^{t+1\left(\mathrm{store}\right)}=\left\{\begin{array}{ll} {\vec{X}}_{i}^{t}+\mu \cdot \left({\vec{X}}_{\mathrm{best}}^{t}-{\vec{X}}_{i}^{t}\right)\cdot \left|\lambda \right|+r_{1}\cdot \left({\vec{X}}_{A}^{t}-{\vec{X}}_{B}^{t}\right) & \mathrm{if}\;\tau_{1}<\tau_{2}\\ \left\{\begin{array}{ll} {\vec{X}}_{\mathrm{best}}^{t}+\mu \cdot \left({\vec{X}}_{A}^{t}-{\vec{X}}_{B}^{t}\right) & \mathrm{if}\;\tau_{1}<\tau_{3}\\ {\vec{X}}_{\mathrm{best}}^{t}\cdot I & \end{array}\right. \end{array}\right.$$

where ${\vec{X}}_{i}^{t+1\left(\mathrm{store}\right)}$ is the new storage position of individual i in the storage area at t+1, ${\vec{X}}_{\mathrm{best}}^{t}$ is the position of the best individual in the population at t, and I is a linearly decreasing factor from 1 to 0.

#### 2.3.2. Retrieval Stage

The retrieval stage and the foraging stage have completely different operational strategies. The cache search and cache recovery strategy are based on the reference point of the nutcracker. In short, there are multiple reference points chosen by the nutcracker to remember where to store food.

- Exploration sub stage: winter search cache

Nutcrackers not only randomly search for their storage, but also use the area near the storage to determine its location. At this stage, the nutcracker may find a storage item at the current cache search location. When no storage item is found, the nutcracker will also develop a promising area around the first reference point to find the storage item. At this point, the mathematical expression of the nutcracker’s behavior is shown in the below equation:

(16) $${\vec{X}}_{i}^{t+1}=\left\{\begin{array}{ll} {RP}_{i}^{(m),t} & \;\;\;\;\;\;\mathrm{if}\;f\left({\vec{X}}_{i}^{t}\right)<f\left({RP}_{i}^{(m),t}\right)\\ {\vec{X}}_{i}^{t} & \;\;\;\;\;\;\mathrm{otherwise} \end{array}\right.$$

where ${RP}_{i}^{(m),t}$ is the *m*-th reference point of the nutcracker’s current position in the current iteration t.

- Development sub-stage: cache recovery

The development sub-stage occurs in spring when the nutcracker begins to search for stored pine nuts. Nutcrackers use spatial memory strategies to locate their storage. After gaining sufficient experience in selecting suitable reference points, the nutcracker will update the storage location based on the appropriate reference points. The formula for updating the position of the nutcracker during the cache search phase is shown below:

(17) $${\vec{X}}_{i}^{t+1}=\left\{\begin{array}{ll} {\vec{X}}_{i}^{t} & \mathrm{if}\;\tau_{3}<\tau_{4}\\ {\vec{X}}_{i}^{t}+\tau_{1}\cdot \left({\vec{X}}_{\mathrm{best}}^{t}-{\vec{X}}_{i}^{t}\right)+\tau_{2}\cdot \left({RP}_{i}^{(m),t}-{\vec{X}}_{B}^{t}\right) & \mathrm{otherwise} \end{array}\right.$$

**Figure 1:**
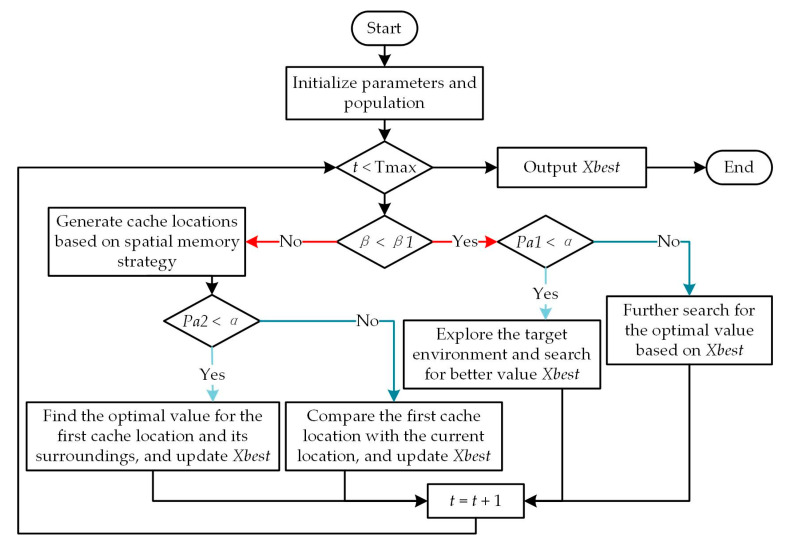
Flowchart of the NOA; the red “Yes” branch indicates the foraging stage, the red “No” branch represents the retrieval stage, the light blue arrow represents the exploration stage, and the blue arrow represents the development stage.

The strategy transformation of the algorithm is shown in Figure 1, where *β*, *β1*, and *α* represent random numbers distributed in the interval [0,1]. The probability parameter *Pa1* undergoes linear decay from 1 to 0 across generations, while *Pa2* remains fixed at 0.2 throughout the optimization process. As illustrated in the flowchart, the algorithm begins with parameter initialization and proceeds through conditional branches governed by these parameters. The *β* and *β1* values determine the forage–retrieve balance, while comparisons with α control the activation of different search strategies, including cache exploration and development.

### 2.4. Model Updating Procedure Integrating the Response Surface Method and the Nutcracker Optimization Algorithm

A new FE model correction method using a bridge health monitoring system is proposed. The first step is to construct an FE model, and the second step is to construct a response surface function that can replace the FE model. The third step is to construct the objective function, and the overall optimization goal is to minimize the function so that the optimized output response value is closer to the real value [38]. The objective function is defined as follows:

(18) $$f_{\min}=\sum_{i=1}^{n}{\left(\frac{f_{\mathrm{si}}-f_{\mathrm{ji}}}{f_{\mathrm{si}}}\right)}^{2}$$

where $f_{\mathrm{si}}$ denotes the experimental frequency of the bridge, and $f_{\mathrm{ji}}$ represents the frequency calculated by the RSM.

**Figure 2:**
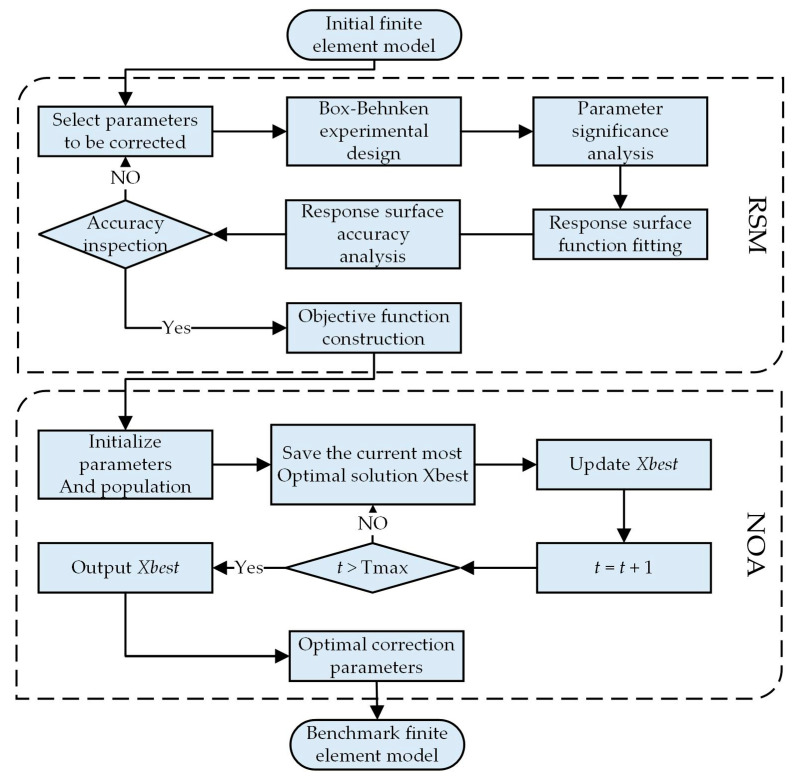
Flowchart of FE model updating based on the RSM–NOA.

Figure 2 illustrates the flowchart of finite element model updating combining RSM and NOA. The process begins with an initial finite element model, followed by the selection of parameters requiring correction. A Box–Behnken experimental design is then employed to establish an efficient sampling scheme for parameter space exploration. Subsequent parameter significance analysis identifies highly significant parameters, while rigorous fitting procedures construct the response surface function, with accuracy verification ensuring the reliability of the surrogate model. Upon successful accuracy validation, the framework proceeds to the optimization phase, where the NOA initializes its population and parameters. The algorithm iteratively searches the parameter space through its distinctive foraging-inspired mechanisms, progressively updating the optimal solution *Xbest* until reaching the maximum iteration count Tmax. The obtained optimal model parameters are subsequently implemented in the finite element model, completing the model updating process.

## 3. Case Study

### 3.1. Comparison of Operational Bridge Frequency Identification

This study employs a municipal bridge as the engineering case. The main bridge comprises a concrete trough-shaped girder with spans of 49.9 m + 104.983 m + 49.9 m and a steel tube concrete arch with a calculated span of 104.983 m. The deck features a reverse S-shaped alignment. The arch ribs, formed as concrete-filled steel tubes with a parabolic profile, have a design rise of 23.33 m and a rise-to-span ratio of 1:4.5. The arch ribs utilize a constant-height, dumbbell-shaped cross-section and are positioned 12.2 m from the transverse centerline. Seven wind braces connect the arch ribs. Eleven pairs of hangers are arranged along the bridge, spaced at 8 m intervals. The upper ends of the hangers are anchored to tensioning bases on the top flange of the arch ribs, while the lower ends are fixed to bases beneath the cross girders.

**Figure 3:**
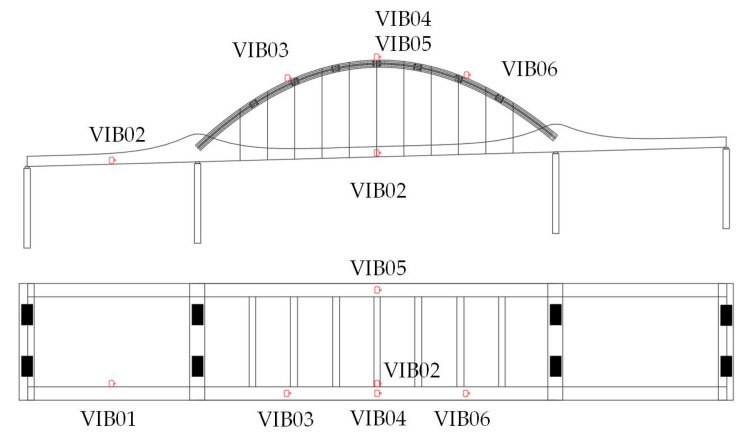
Arrangement of acceleration sensors.

The data in this case study were obtained from the long-term health monitoring system of a continuous beam–arch composite bridge. The system employs accelerometers installed at critical locations to continuously record structural vibration responses, providing a benchmark for modal parameter identification and model updating. The bridge monitoring system consists of six measurement points, located at the mid-span of the left-side main girder, the mid-span of the central main girder, and the quarter and three-quarter points of the arch ribs, ensuring comprehensive coverage of the primary vibration-sensitive regions of the structure. The sensor arrangement is depicted in Figure 3.

**Figure 4:**
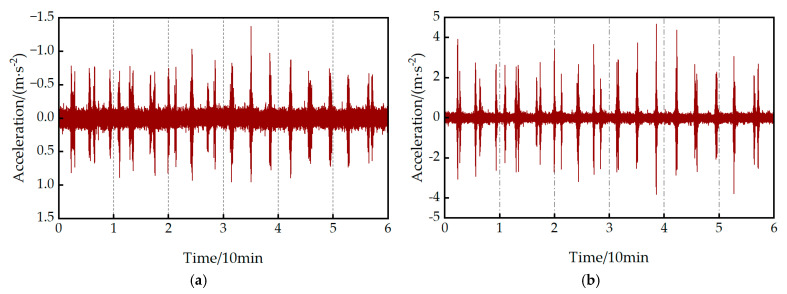
Preprocessed sensor acceleration time history curve: (**a**) diagram of lateral vibration; (**b**) diagram of vertical vibration.

One hour of vibration data from an acceleration sensor (VIB02) at the mid-span cross-section on August 16th was selected as the analysis sample. Given that the railway traffic load aligns with the white noise statistical properties required by SSI, one hour of data during train passage was used as the raw dataset. Due to the necessity of eliminating temperature effects [39,40], wavelet decomposition was applied to remove trend items from the raw lateral and vertical vibration data, as illustrated in Figure 4. To ensure the accuracy of structural modal identification, peak picking theory and SSI are combined to analyze vibration response signals.

**Figure 5:**
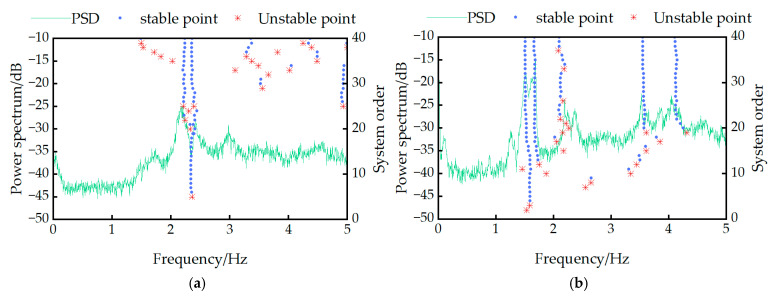
Stability graph of stochastic subspace identification: (**a**) lateral vibration frequency; (**b**) vertical vibration frequency.

Figure 5 clearly shows prominent peaks in both lateral and vertical power spectral density (PSD) curves. According to the peak picking theory, the horizontal coordinates corresponding to these peaks represent the frequencies identified by the peak-picking method. Simultaneously, the horizontal coordinates of the stable axis formed by stationary poles in these figures indicate the frequencies identified by SSI. Notably, a consistent coincidence exists between the positions of the stable axis and the spectral peaks. This congruence demonstrates the high reliability of the bridge modal frequencies identified from vibration monitoring data acquired by the structural health monitoring system. Based on this analysis, the identified vertical modal frequencies are 1.511 Hz and 1.684 Hz, while the lateral modal frequencies are 2.187 Hz and 2.422 Hz.

### 3.2. Finite Element Model of the Continuous Beam–Arch Composite Bridge and FE Model Results

**Figure 6:**
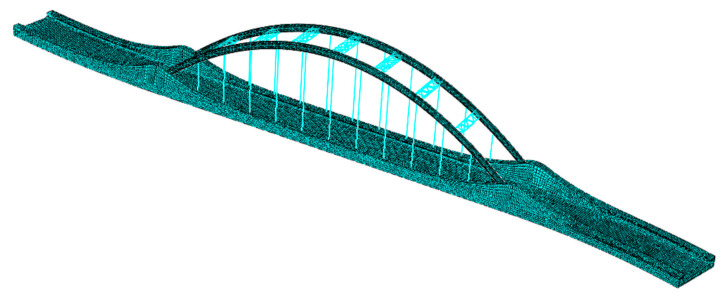
FE model of the bridge structure.

**Figure 7:**
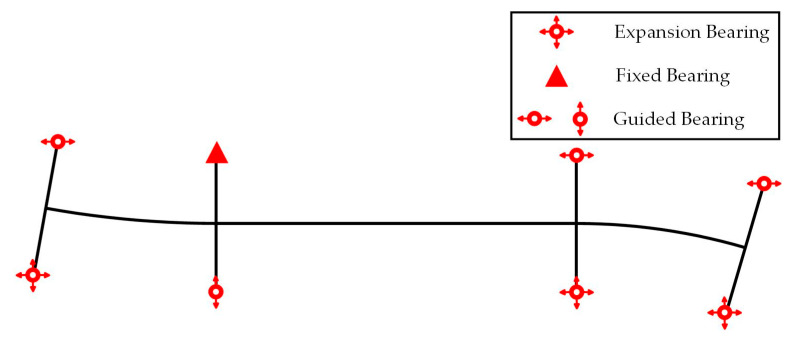
The locations and types of supports in the completed state.

A FE model of a continuous beam–arch composite bridge was established using ABAQUS 2024 software. The main girder and bearings were modeled using beam elements, while the steel tubular main arch ribs and wind bracings were simulated with shell elements. The bridge hangers were represented by truss elements. To enhance the model’s fidelity, the material properties of all components were defined based on actual project data, as detailed in Table 1. In the ABAQUS model, rigid constraints were applied to the bearings to ensure accurate interactions between bridge components, and the connection between the bearings and the main girder was established using tie constraints. The connection surfaces between the tied-arch and hangers with the main girder were constrained using embedded regions, while the cross-sections of the arch body and the arch axis were connected via tie constraints. The wind bracings and arch body, as well as the hangers and arch body, were coupled using coupling constraints. Figure 6 illustrates the three-dimensional representation of the established FE model. To ensure that the FE model accurately replicated the constraints of the actual bridge, the boundary conditions at the supports precisely reproduced the constraints under the completed state of the real bridge [41,42]. Different constraints were applied beneath the bearings, as shown in Figure 7. The choice of meshing strategy plays a critical role in the accuracy of the FE model [43]. Therefore, a structured meshing approach was adopted in this study, where solid elements were discretized using fully hexahedral elements, shell elements were meshed with triangular elements, and truss elements were uniformly distributed.

Modal parameters were extracted from the initial FE model using the Lanczos algorithm, with calculation results presented in Table 2. The frequency deviation for the first-order lateral bending mode (H1) was 3.6%, while this error increased to 8.3% for the second-order lateral bending mode (H2). Similarly, the first-order vertical bending mode (V1) exhibited an error of 3%, and the second-order vertical bending mode (V2) showed an error of 7.4%. The observed reveals an average error of 5.58%, with deviations exceeding 7% for higher-order modes (H2 and V2), compellingly demonstrating the necessity of FE model updating.

### 3.3. Construction of the Response Surface Model

Based on engineering experience, eight material parameters—the mass density and elastic modulus for the main girder, main arch, wind bracings, and hangers—were initially selected as candidate updating parameters. Subsequently, the forward difference method within finite difference approximation was employed to conduct a sensitivity analysis on these candidate parameters. This analysis utilized an incremental step size of 20% relative to their initial values to further identify parameters exhibiting high sensitivity, which were then designated as the final updating parameters.

**Figure 8:**
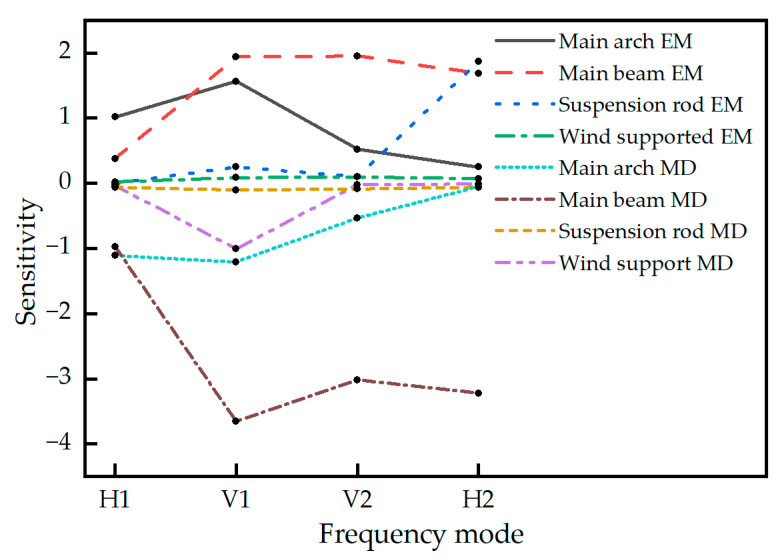
Sensitivity analysis. EM represents the mass density; MD represents the elastic modulus.

As illustrated in the sensitivity analysis results shown in Figure 8, both the elastic modulus (E1) and mass density (D1) of the main girder demonstrated high sensitivity to the first-order vertical frequency, second-order vertical frequency, and second-order lateral frequency, while showing relatively lower sensitivity to the first-order lateral frequency. The elastic modulus (E2) and mass density (D2) of the main arch exhibited high sensitivity to the first-order lateral frequency and first-order vertical frequency, moderate sensitivity to the second-order vertical frequency (less pronounced than for the first-order modes), and low sensitivity to the second-order lateral frequency, with the mass density exhibiting negligible sensitivity to the latter. The elastic modulus of the hangers (E3) showed negligible sensitivity to the first-order lateral frequency and both first- and second-order vertical frequencies but displayed significant sensitivity to the second-order lateral frequency. Conversely, the mass density of the hangers (D3) exhibited high sensitivity to the first-order vertical frequency but negligible sensitivity to other frequencies. The elastic modulus and mass density of the wind bracings demonstrated negligible sensitivity across all modal frequencies.

Consequently, the elastic modulus and mass density of the main girder, the elastic modulus and mass density of the main arch, and the elastic modulus and mass density of the hangers were ultimately selected as the updating parameters. The permissible adjustment range for each parameter was constrained to ±30% of its initial value to preserve physical plausibility.

**Figure 9:**
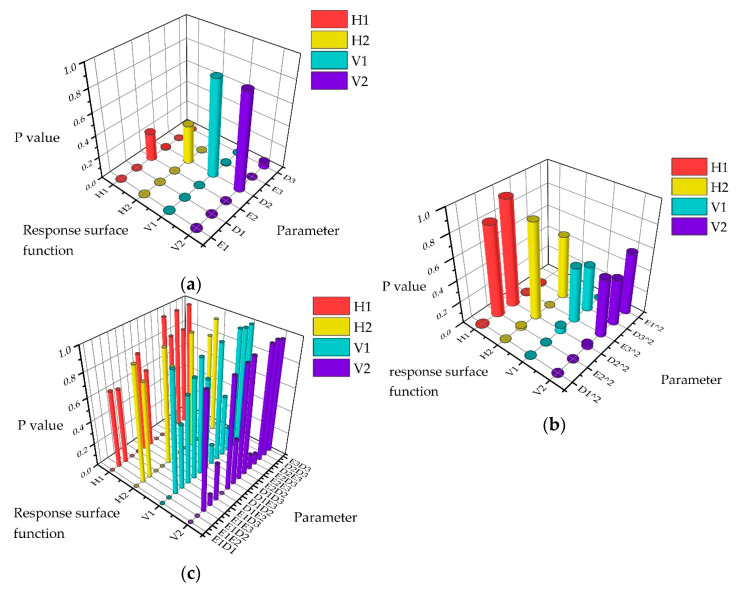
Significance analysis for parameters: (**a**) primary; (**b**) quadratic; (**c**) interaction.

This case utilized four modal frequencies obtained from the structural health monitoring system as the target responses. The Box-Behnken experimental design method was employed to construct the parameter sample space, with Design-Expert 13 software generating 54 sets of parameter combinations. Significance analysis of the sample parameters was performed using the F-test method at a significance level of α=0.05, with the results depicted in Figure 9. A complete quadratic polynomial was adopted to establish the parameter-frequency RSM. The regression coefficients were solved via the least squares method, yielding an explicit mathematical relationship between the input parameters and output responses. Validation through two key metrics, the coefficient of determination ($R^{2}>0.99$) and the root mean square error (RMSE<0.001), as presented in Table 3, demonstrates that the RSM accurately characterizes the parametric frequency mapping relationship of the FE model. The relative error was below 0.1%, satisfying the accuracy requirements for substituting the FE model in iterative updating procedures.

### 3.4. Optimization Algorithms and Model Updating Results

We incorporated the response surface function and objective function into the NOA. All calibration parameters can only vary within ± 30% of the initial value to maintain physical rationality. We used the NOA algorithm to iteratively search the RSM and find the optimal correction parameters that minimize the objective function.

The optimized parameters obtained using the NOA algorithm are presented in Table 4. Concrete material parameters demonstrate minor variations, whereas significant modifications are observed for steel and hanger materials. Subsequent validation through FE model simulation incorporating these optimized parameters reveals natural frequency errors after correction in Table 5. Compared to pre-updated values, all-natural frequencies exhibit reduced errors except for the fundamental lateral bending mode frequency, which shows increased deviation.

**Figure 10:**
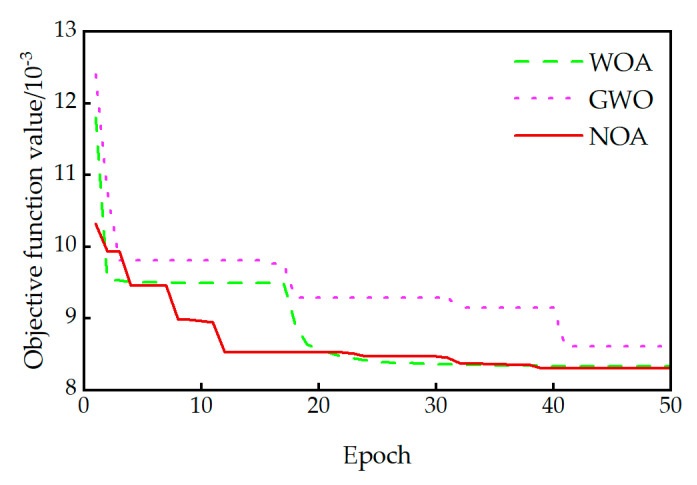
Convergence of the objective function under different optimization algorithms.

To rigorously evaluate the performance of the NOA algorithm, its results were compared against those obtained using the whale optimization algorithm (WOA) and the grey wolf optimizer (GWO). All three optimization algorithms employed identical configuration settings: an initial population size of 30 and a maximum iteration limit of 50. The convergence characteristics of these algorithms are illustrated in Figure 10. The NOA, WOA, and GWO algorithms converged toward the vicinity of the objective function’s minimum value after approximately 13, 21, and 41 iterations, respectively. Notably, the NOA algorithm demonstrated the fastest convergence rate.

The comparative analysis of frequency errors in Table 5 demonstrates that post-correction frequency errors from all three algorithms remained below 7.1%. However, all algorithms exhibited larger errors specifically for the first-order lateral bending mode calculation. This phenomenon may arise from a compromise effect inherent in balancing the corrections required for the second-order vertical bending and second-order lateral bending modes. Detailed results confirm that the NOA algorithm achieved a significant comprehensive advantage in correcting the first-order lateral frequency, yielding an error of only 3.8%. This performance is substantially superior to the corresponding error values of 6.9% from the GWO algorithm and 7.1% from the WOA algorithm.

## 4. Conclusions

This paper proposes an FE model updating framework integrating the RSM and the NOA, addressing the discrepancies between FE models and actual structures. The main conclusions are summarized as follows:

1. The SSI method was employed to analyze the operational vibration response of an in-service municipal bridge, successfully extracting its second-order vibration frequencies. These experimentally identified frequencies served as the benchmark for subsequent model updating;
2. The NOA features dual exploration strategies, enhancing its global optimum localization. Integrating RSM with NOA accelerated parameter identification compared to standalone methods. Experimental results confirmed that the NOA outperforms GWO and the WOA in convergence rates and enhanced model accuracy;
3. Experimental validation indicates that the structural frequencies extracted from the updated FE model by the proposed method exhibit improved consistency with the frequencies identified from the structural health monitoring data, demonstrating the method’s effectiveness in enhancing numerical model accuracy. Moreover, the proposed RSM–NOA method reduced the average frequency error from 5.58% to 2.75% by updating the model parameters.

In summary, this study validates the RSM–NOA framework for finite element model updating of a continuous beam–arch composite bridge. While effective for this application, the method’s performance on structures with highly nonlinear material behavior requires further investigation. Future work will explore extensions to more complex structural systems.

## Figures and Tables

**Table 1:** Bridge material parameters.

Material	Mass Density/kg·m^−3^	Elastic Modulus/MPa	Poisson’s Ratio	Structural Component
C55 concrete	2.55 × 10^3^	3.55 × 10^4^	0.2	Main girder, steel tube infill
Steel	8.0055 × 10^3^	20.6 × 10^4^	0.3	Arch rib, wind bracing, bearing
Steel strand	8.0055 × 10^3^	19.5 × 10^4^	0.2	Hanger cables

**Table 2:** Comparison between measured frequencies and theoretical frequencies.

Vibration Mode	FEA Calculated Value/Hz	Measured Value/Hz	Error/%
Vertical Bending 1 (V1)	1.465	1.511	3
Vertical Bending 2 (V2)	1.567	1.684	7.4
Horizontal Bending 1 (H1)	2.108	2.187	3.6
Horizontal Bending 2 (H2)	2.22	2.422	8.3

FEA: Finite Element Analysis.

**Table 3:** Fitting accuracy of the RSM.

Frequency	H1	H2	V1	V2
$R^{2}$	0.9998	0.9996	0.9983	0.9998
RMSE	0.00021	0.00032	0.00074	0.00059

**Table 4:** Update of parameters via the NOA.

Parameter/Unit	Pre-Correction	Post-Correction	Change Rate/%
E1/MPa	35,500	33,122.78	6.7
D1/kg·m^−3^	2.55 × 10^3^	2.48 × 10^3^	2.7
E2/MPa	195,000	151,841.69	22.1
D2/kg·m^−3^	8.0055 × 10^3^	10.4 × 10^3^	29.9
E3/MPa	206,000	179,298.83	12.9
D3/kg·m^−3^	8.0055 × 10^3^	9.45 × 10^3^	18

**Table 5:** The error between theoretical frequencies and measured values across optimization algorithms.

Mode	Measured Value/Hz	GWO Correction		WOA Correction		NOA Correction	
		Calculated Value/Hz	Error/%	Calculated Value/Hz	Error/%	Calculated Value/Hz	Error/%
V1	1.511	1.501	0.6	1.508	0.2	1.505	0.3
V2	1.684	1.765	4.8	1.757	4.3	1.763	4.6
H1	2.187	2.036	6.9	2.03	7.1	2.103	3.8
H2	2.422	2.475	2.2	2.467	1.9	2.477	2.3

## Data Availability

The data presented in this study are available on the request from the corresponding author.

## Abbreviations

The following abbreviations are used in this manuscript:

FE	Finite Element
RSM	Response Surface Model
NOA	Nutcracker Optimization Algorithm
SSI	Stochastic Subspace Identification
OMA	Operational Modal Analysis
PSD	Power Spectral Density
FEA	Finite Element Analysis
GWO	Grey Wolf Optimizer
WOA	Whale Optimization Algorithm
